# Climate Change Impacts on Disaster and Emergency Medicine Focusing on Mitigation Disruptive Effects: an International Perspective

**DOI:** 10.3390/ijerph15071379

**Published:** 2018-07-01

**Authors:** Daniel Aiham Ghazali, Maximilien Guericolas, Frédéric Thys, François Sarasin, Pedro Arcos González, Enrique Casalino

**Affiliations:** 1 Emergency Department and EMS, University Hospital of Bichat, Paris 75018, France; maximilien.guericolas@aphp.fr (M.G.); enrique.casalino@aphp.fr (E.C.); 2 Ilumens Simulation Center, University of Paris-Diderot, Paris 75018, France; 3 Acute Care Division & Emergency Department, Grand Hôpital de Charleroi, Charleroi 6040, Belgium; frederic.thys@uclouvain.be; 4 Faculty of Public Health & Medicine, Catholic University of Louvain, Brussels 1348, Belgium; 5 Emergency Department, University Hospital of Geneva, Geneva 44041, Switzerland; francois.sarasin@hcuge.ch; 6 University of Geneva Medical School, Geneva 1205, Switzerland; 7 Unit for Research in Emergency and Disaster, Department of Medicine, University of Oviedo, Oviedo 33006, Spain; arcos@uniovi.es; 8 University of Paris Diderot, Sorbonne Paris Cité, EA 7334 Recherche clinique coordonnée ville-hôpital, Méthodologies et Société (REMES), Paris 75018, France; 9 Study Group for Efficiency and Quality of Emergency Departments and Non-Scheduled Activities Departments, Paris 75018, France

**Keywords:** climate change, emergency medicine, health, disaster preparedness, management, European perspective

## Abstract

In recent decades, climate change has been responsible for an increase in the average temperature of the troposphere and of the oceans, with consequences on the frequency and intensity of many extreme weather phenomena. Climate change’s effects on natural disasters can be expected to induce a rise in humanitarian crises. In addition, it will surely impact the population’s long-term general health, especially among the most fragile. There are foreseeable health risks that both ambulatory care organizations and hospitals will face as global temperatures rise. These risks include the geographic redistribution of infectious (particularly zoonotic) diseases, an increase in cardiac and respiratory illnesses, as well as a host of other health hazards. Some of these risks have been detailed for most developed countries as well as for some developing countries. Using these existing risk assessments as a template, organizational innovations as well as implementation strategies should be proposed to mitigate the disruptive effects of these health risks on emergency departments and by extension, reduce the negative impact of climate change on the populations they serve.

## 1. Introduction 

Over the past sixty years, climatologists have witnessed climate change due to global warming, which has led to an increase in average oceanic and atmospheric temperatures. Current scientific consensus attributes this to multiple phenomena among which human (anthropogenic) activity is prominent [1,2]. The human impact is linked to the accumulation of carbon dioxide and other pollutant gas emissions resulting from the use of fossil fuels, in turn responsible for an added greenhouse effect which has increased the temperature of the troposphere. In the absence of significant mitigating measures, its predicted evolution in the coming decades is worrying [3].

Global warming is associated with other climate phenomena. The most prominent phenomenon is an exacerbation of the El Niño phenomenon, which affects wind patterns, sea temperatures, and precipitation. El Niño and La Niña correspond to the two opposite phases of the coupled ocean/atmosphere phenomenon called El Niño/Southern Oscillation (ENSO) [4]. ENSO has a planetary impact [5] increased by global warming in terms of frequency and intensity [6]. This phenomenon, a source of origin of specific natural disasters, has already given rise to situations of humanitarian and health crises (e.g., in Haiti, Japan, and so forth). In addition to these situations that are at a city, regional, national, or even continental scale, climate change directly affects the health of the involved populations, especially the most vulnerable [7]. Environmental and meteorological changes impact air, water (drinking), and food quality and may be the greatest threat to the health system in crisis situations [8]. The direct consequence will be increased health risks with greater vulnerability and insecurity of the population’s water and food access, as well as a modified distribution of pathogens and associated vectors. These risks will be exacerbated with an increased frequency of extreme climatic events and would be compounded by increased migration phenomena and changes in agricultural production, ultimately resulting in a risk of undernutrition and civil disruption for a large part of the world population [5,9,10].

All countries of the world will face a risk to their health care system. However, risks and their consequences are likely to be lessened in developed countries because of a more robust response capacity to adapt to these potential new risks [8]. Nevertheless, all of this depends on the ability to identify risks, organize response systems, and put in place procedures that reduce risks and their impact on the population [4]. Around the world, there are great disparities in hospital response, namely the percentage of countries’ health expenditure, bed density, the size and surface area of hospitals, the status of hospitals (public and private), hospital organization, and funding. Total health expenditure ranges from less than 1% to more than 17% of gross domestic product depending on the country. Around 40% of this goes to hospitals in developed nations. These differences already have a real impact on nonscheduled care (emergencies) on a daily basis. Climate change and disasters are likely to significantly highlight these disparities. In addition, affected populations may not be culturally prepared not only for these climatic changes, but also for intrinsic or external immune levels (vaccinations). 

Emergency caregivers are the first actors in the case of natural disaster or in the case of an increased influx of patients in the hospital with a quasi-systematic arrival in ED (emergency department). Consequently, emergency medicine and disaster medicine should be prepared to be active players in the planning to prevent and fight against the new risks linked to climate change. Based on the literature, the aim of this study is to identify the impacts of climate change on health and more specifically, on EDs. In light of the effects of climate change, specific strategies will be proposed to implement now and moving forward for EDs in Europe and internationally (especially in the less developed countries).

## 2. Background: How Climate Change Is Impacting on Health in General

### 2.1. The Direct Effects of Increased Heat on People Leading to Heightened Risks of Cardiovascular Disease, Respiratory Disease, and Renal Disease

In developed countries, there have been several studies on the specific effects of extreme temperatures on stroke and cardiovascular events [11]. A study conducted in 12 U.S. cities showed that the effects of temperature on hospital admissions predominantly occur within a few days after exposure [12]. Low temperatures cause blood vessels to narrow, which increases blood pressure and therefore the risk for stroke and other cardiovascular events [13]. High temperatures cause blood vessels to dilate, which increases cardiac output and associated risks of decompensated heart failure [14]. Extreme temperatures cause stress on the cardiovascular system, with a higher risk of coronary diseases, especially in the elderly [15]. In Vietnam, it was demonstrated that the average point of minimum admissions for cardiac diseases was at 26 °C. Rates of admission increased below this temperature point [16]. 

On respiratory implications, it has been shown that the frequency of ED admissions is increased for asthma and chronic obstructive pulmonary disease (COPD) with an increase in pollution rates [17]. This relates to climate change as it has been shown to increase pollution. Many climate change effects are considered to have negative repercussions on respiratory health and to enhance the frequency and severity of respiratory diseases such as asthma in the general population [18]. Global warming is expected to affect the start, duration, and intensity of the pollen season on the one hand, and the rate of asthma exacerbations due to air pollution, respiratory infections, and/or cold air inhalation, as well as other respiratory conditions, on the other hand [18]. Furthermore, among older adults, a recent and detailed study of the etiological climatic factors demonstrates that short-term exposure to ambient PM2.5 (particulate matter <2.5 microns in diameter) is associated with more ED visits and hospitalizations for pneumonia, severe pneumonia, increased mortality, and increased health care costs. Exposure to other air pollutants (NO_2_ and O_3_) modestly increases pneumonia risk and illness severity [19].

Because of the rise in temperatures, there is an increase in acute kidney injuries and in chronic kidney diseases consistent with heat stress nephropathy [20]. This type of renal disease is different from those due to glomerulonephritis, diabetes, or hypertension, but is a detrimental factor for those diseases as well. 

### 2.2. The Effects of Heat Waves on General Health

The impact of extreme temperature variations on health has been documented in several international studies [21,22], such as those carried out in North America [23,24], Europe [25,26], Asia [27,28], and Australia [29,30]. They all describe a significant effect of heat waves on mortality and morbidity. The effects of heat waves on people are mediated by dehydration, both directly through heat stress and through exacerbation of cardiovascular, cerebrovascular, renal, and respiratory illnesses. A Chinese study on heat-related illness described symptoms in cases of mild and severe illness [31]. Heat waves were defined as seven or more consecutive heat days with a maximum temperature over 35 °C. A positive association between maximum temperatures and occurrence of heat-related diseases was apparent. Mild illness symptoms are dizziness, headache, flushing, thirst, profuse sweating, weakness, palpitation, rapid pulse, attention deficit, loss of coordination, and body temperature ≥38.5 °C. Severe illness symptoms are heat stroke (including hot and dry skin, disturbance of consciousness, body temperature ≥40 °C), heat cramps (including muscle spasms with pain, usually in the calves, arms, abdominal wall, and back; altered consciousness; normal body temperature), and heat exhaustion (headache, sweating, thirst, nausea, vomiting, moist and cool skin, drop in blood pressure, tachycardia, mild dehydration, normal or slightly high body temperature).

### 2.3. The Health Impact of Increased Likelihood of Climatic Disasters

Climate change has led to an increase in the frequency of violent phenomena such as the hurricanes and tropical storms that recently affected the Caribbean (Hurricanes Irma and Maria in September 2017). During periods of heavy rains and droughts, floods expose populations to the risk of drowning and traumatic pathologies: bruises, cuts or sprains, burns, electrocutions, snake bites, and wound infections [4]. The impact of the increase in traumatic pathologies has been described in tropical regions exposed to annual seasonal variations in rainfall. Both floods and droughts can coexist and succeed each other over time, increasing the risk of flooding and landslides. It is estimated that the frequency and intensity of storms and other occasional weather phenomena (very heavy rainfall and tornadoes, for example) increases with climate change. Areas that might seem less exposed to these phenomena, such as Europe, will be increasingly affected. Thus, it is estimated that in Europe, this risk will increase significantly in relation to climate change, and that mortality from traumatic causes may be significant [32]. In 2018, it is estimated that these phenomena currently cause over 60,000 deaths annually, with predictions of more than 250,000 annual deaths between 2030 and 2050 [33].

### 2.4. Changes in Infectious Disease Epidemiology Directly as a Result of Microbiological Changes

Human activity, directly and through the effects of induced climate change, is linked to changes in the ecosystem, such as changes in vector distribution, population susceptibility, and population exposure [34]. Climate change will also promote the embedding and development of invertebrate vectors. The risks for malaria, dengue and dengue hemorrhagic fever, yellow fever, chikungunya, Zika virus, West Nile fever, Lyme disease, and leishmaniasis will be increased in currently endemic areas, but also in areas that are currently free of malaria and these other infections [35]. Rodent-borne diseases may proliferate in this context, such as leptospirosis [36] and Hantaan virus [37]. Epidemics of these pathologies have been reported following floods in Europe [38]. The spread of arthropod vectors (e.g., Hantaan virus, Seoul virus) to previously unexposed cities due to climate change has already been reported [37]. The re-emergence of certain diseases such as malaria in formerly malarious areas, such as in southern Europe, has been mentioned due to the rise in temperatures [39]. The pathologies involved may present themselves as severe clinical forms requiring admission to intensive care units [40].

### 2.5. The Population Impacts Associated with Major Shifts of Population Due to Rising Sea Levels and Disturbances to Food and Water Supplies

The changes in infectious disease are also indirectly linked to changing vectors patterns and increased risks of food- and waterborne diseases. Rising temperatures, flooding events, and abnormal water accumulation as a result of rainfall and stream overflows will result in changes in habitats, hibernation patterns, and the reproduction cycles of reservoir species. Additionally, migratory flows linked to climate change [9] will also modify the geographic distribution of susceptible populations [41]. This will promote the emergence of re-emerging or exotic zoonotic diseases in nonendemic areas [42]. Moreover, in the regions that will present difficulties in ensuring access to drinking water, human populations and livestock will be exposed to diarrheal diseases [43,44]. Thus, we can speculate a rise in bacterial, viral, and parasitic infections affecting humans (viral hepatitis A, enteropathogenic *Escherichia coli*, *Campylobacter*, salmonellosis, rotavirus) [4]. In South Korea, incidences of waterborne and foodborne diseases rose after meteorological disasters, as was measured over 65 floods and typhoons between 2001 and 2009 [45]. It appears that the incidences of *Vibrio vulnificus* septicemia and shigellosis were associated with meteorological disasters. Added to this situation is an increased risk, in occidental countries, of exposure to imported agents such as cholera [46] and zoonotic diseases such as cryptosporidium [47].

### 2.6. The Mental Health Consequences Associated with Many of the Above Phenomena as Well as Direct Heat Effects

Climate change-induced extreme weather events are expected to increase post-traumatic stress disorders [3,48]. The geographic distributions of suicidality and of extreme climate events have been demonstrably linked; therefore, the effects of climate change are predicted to favor an increase in suicide rates [49,50]. In a recent study, ambient air particle concentrations seemed to be associated with the number of visits to the psychiatric emergency unit during the warm season in Sweden [51]. Population displacement is another hardship that is predicted to rise in frequency because of climate change. The demographic and socioeconomic impacts of an increase in forced population displacements would surely result in higher rates of stress-related psychiatric disorders [52]. Additionally, different countries show heterogeneous levels of preparedness to provide the necessary long-term assistance to potentially affected populations [53].

## 3. Specific Risks of Climate Change to EDs

### 3.1. Increased Demand Generally

Global warming has an impact on short-term population health status and on emergency response structure functionality. The increased risks associated with climate change represent an emerging threat to the health status of affected communities [54]. The impact of climate change on population health and on health care systems of developed countries in Europe and North America has been assessed and recognized as a major health risk [8,32,33,48,55,56]. Global warming will result in an increased frequency and severity of cardiac diseases and COPD [57]. A recent study analyzed ambient temperature and added heat wave effects on hospitalizations in California from 1999 to 2009 [58]. Positive associations with heat waves were found for acute renal failure, dehydration, respiratory disease, and respiratory disease with a secondary diagnosis of diabetes. For temperature, positive associations were observed for acute renal failure, appendicitis, dehydration, ischemic stroke, mental health, noninfectious enteritis, and primary diabetes. 

### 3.2. Climate Changes and Disaster Emergency Medicine

Observations since 1950 indicate increases in some forms of extreme weather events. Reports on Extreme Events and Disasters (SREX) by the Intergovernmental Panel on Climate Change (IPCC) predicts further increases, including a growing frequency of heat waves, tropical cyclone intensity, and increasing intensity of droughts, rain, and floods [59]. Additionally to natural disasters, states have had to deal with the management of serious to minor casualties. A massive influx of these injured affected hospitals and healthcare facilities with an undeniable impact on the management of patient flows associated with emergency departments (EDs). In spite of existing plans for crisis management, developed countries will also be affected by these unusual phenomena. Thus, Florida and its EDs were also affected by the last hurricanes. Other phenomena also affect the global health system. The management of phenomena of this magnitude would require national or even international levels of intervention to support the affected countries. Habitually, this scale of management relies on military and civilian actors in emergency and humanitarian medicine. Although the management of such phenomena cannot be the sole responsibility of existing hospitals and of their EDs, the United Nations (UN) and the World Health Organization (WHO), as well as national organizations, will rely on available resources and healthcare personnel to respond to humanitarian disasters. There is also a need to strengthen support for hospitals and health care facilities in the least developed countries. Sometimes, it is a country that has to help its local region when the victim of a natural disaster, when a neighboring state is incapable of fighting alone against the sudden onset of a disaster. France, when faced with the violence of hurricane Irma, in September 2017, mobilized more than 400 local health professionals as well as tons of equipment to support the local health network in Saint-Martin (an island in the northeast Caribbean Sea). Thus, all hospitals could consider a building a system for rapid, flexible deployment of human and material means to better respond to the increase in natural disasters. In addition, it is important that the entire care network upstream and downstream of the hospital participates in this vigilance. The concept of a sentinel network in all its components is probably the best guarantee of rapid impact identification and the structured implementation of an adequate care response.

### 3.3. Outreach Requirements 

It is obvious that the whole population must be aware of the importance of the problem, but also of the disastrous consequences of climate change on the environment, the economy, and the security of the population. In many countries, public authorities and scientific societies have established regional, national, and even continental risk maps. This step is essential to the preparation of response plans adapted to the identified risks. All actors in the health system should participate in the development of risk or hazard mapping, and promote methods to reduce the impact of the identified risks on the population and in particular, on the most fragile populations [60]. 

For EDs, an emergency checklist has been proposed [61]:

1. Emergency plan: Based on a risk assessment, develop an emergency plan using an all-hazards approach focusing on capacities and capabilities.
2. Policies and procedures: Develop and implement policies and procedures based on the plan and risk assessment.
3. Communication plan: Develop and maintain a communication plan that complies with both federal and state law. Patient care must be well coordinated within the facility, across health care providers, and with state and local public health departments and emergency systems.
4. Training and testing program: Develop and maintain training and testing programs, including initial and annual trainings, conducting drills and exercises, or participating in an actual incident that tests the plan.

The WHO Executive Board endorsed a new work plan on climate change and health [62]. This includes:

1. Partnerships: to coordinate with partner agencies within UN system, and ensure that health is properly represented in the climate change agenda.
2. Awareness raising: to provide and disseminate information on the threats that climate change presents to human health, and opportunities to promote health while cutting carbon emissions.
3. Science and evidence: to coordinate reviews of the scientific evidence on the links between climate change and health, and develop a global research agenda.
4. Provide support for implementation of the public health response to climate change: to assist countries to build capacity to reduce health vulnerability to climate change and promote health while reducing carbon emissions.

## 4. The Mitigation Strategies of ED Managers

### 4.1. Capability Development

When it comes to climate change-related pathologies, emergency physicians, including those outside usual endemic areas, should be prepared to recognize and treat infectious diseases that are considered rare locally. Furthermore, especially when new facilities are built, hospitals and EDs must anticipate an increased rate of visits and admissions [57,63]. ED management must also take into account the increased use of emergency response resources in the event of an exceptional variation in ambient temperature. In addition, social network tools specifically adapted to monitoring and sharing pertinent information regarding these health hazards should be set up at the global level. The emergence of new technologies in climate analysis and flow analysis should also be rapidly integrated into this approach. 

### 4.2. Enhanced Surge Capacity

The World Association for Disaster and Emergency Medicine (WADEM) recommends that all disaster and emergency professionals and organizations adopt a risk-based approach to emergency planning that prepares for and enhances resilience to climate change effects, and also recommends linking this to the implementation of the Sendai Framework for Disaster Risk Reduction (2015–2030) [64]. Ambient temperature has been shown to strongly affect emergency care service utilization. Significant increases in mortality and emergency hospital admissions were observed during heat waves, mainly affecting the elderly and people with cardiovascular, renal, or diabetic diseases [29]. Specific monitoring of ambient temperatures and of total suspended particulates by the Ministry of Transportation would therefore help to respond to the increased frequency of high-risk patients consulting EDs [65]. Emergency medical structures should implement the WHO-proposed strategy for managing health crises and put in place policies for the prevention and management of risks [66]. The practice of emergency medicine should include awareness of and preparedness for the risks associated with climate change, particularly targeting situations that could have a direct impact on EDs.

### 4.3. Resources and Equipment Requirements

The recognized risks related to climate change should lead health service administrators to consider programs for continuous improvement of the quality and the level of training of the ED personnel. This would make it possible to cope with even greater increases in ED attendance due to usual pathologies or due to climate-related health crises. Moreover, these programs should make it possible to prevent crisis situations related to rare or not-yet-existent disease epidemics in our environment. 

Emergency services are experiencing a steady and significant increase in their activity and deterioration in their waiting times. ED length of stay has been associated with ED crowding [67], which is associated with decreased quality of care, increased occurrence of side effects, and dissatisfaction of patients and caregivers [68]. We consider that EDs will have to continue to carry out their conventional care missions and in addition, face the newly identified risks brought about by climate change. The challenges we face also represent an opportunity to question our understanding of the usual dysfunctions that plague EDs [69] and to ambitiously redefine ED quality and performance during crises. To this end, it is essential to review our current organizations and to implement quality improvement programs, strategies for simplification of care processes, and tools to reduce ED crowding [70]. Reducing crowding aims to achieve optimal functioning of Emergency Medical System (EMS) and EDs to ensure responsiveness to increasingly frequent and uncharted health crisis situations. 

## 5. European Considerations 

### 5.1. Preparation for the Direct Impacts of Climate Change

Climate change is widely regarded as a contributing factor for natural disasters, for the geographic redistribution of infectious diseases (particularly zoonotic ones), and for the increase in chronic diseases as well as other health hazards. Some of these risks have been highlighted in various reports for most developed countries as well as for some developing countries [54]. Using these risks as a template, organizational innovations as well as novel implementation strategies should be proposed to mitigate the disruptive effects of these health risks on EDs and by extension, reduce the negative impact of climate change on the populations they serve. A growing body of analysis is providing strong evidence to develop and support policies for managing and protecting public health from meteorological disasters [45]. In this context of ongoing preparations to face the impact of climate change, experience gained from previous extreme climate events is already being put to use. For example, in Taiwan, a proper prehospital transportation plan for emergency medical services (EMS), in the event of burdensome numbers of casualties resulting from extreme climate events, was conceptualized after the 88 wind-caused disasters caused by Typhoon Morakot [71]. Developing straightforward facility preparedness plans, with standardized procedures and specific job descriptions, would strengthen responses to future natural disasters [72]. At present, there is a growing tendency to develop courses dealing with complex humanitarian emergencies [73], but this effort should be strengthened because of the rapid climate changes that are observed today. A recent review of literature regarding disaster preparedness provides evidence that there is a lack of readiness for disaster response in the hospitals and EDs that are on the front line to accommodate patients. The findings from this review highlight the benefits of further research and of provisioning for well-grounded disaster exercises that mimic actual events [74]. A recent study of nurse interviews concerning prior disasters suggests that disaster preparedness education in nursing schools and in practice settings should include more hands-on exercises as well as specific policies on nurses’ roles during disasters [75]. Emergency physicians and nurses are expected to provide effective services by using their professional expertise to reduce the risks posed by disasters. Thus, specific competencies are essential for coping with disasters and could be improved through education and training programs that enhance disaster preparedness [76]. Active participation in simulation-based education should help to increase preparedness education. Large-scale disaster simulation exercises should be carried out and repeated every year with multidisciplinary team participation. This hypothesis is reinforced by a recent review that found a lack in training for disaster management, emergency communication, psychological first aid, public health interventions, disaster law and ethics, media handling, and humanitarian responses in an overseas setting [77]. Educational institutions and health and human service organizations must commit to increasing access to a variety of tested disaster-related educational programs for caregivers [78].

### 5.2. Dealing with Climate Refugees

Since 2009, an estimated one person every second has been displaced by a disaster, with an average of 22.5 million people displaced by climate- or weather-related events since 2008 [79]. These populations have been exposed to new risks, mainly zoonoses, and given their socioeconomic living conditions, they have an increased risk of pathologies such as tuberculosis, Human Immunodeficiency Virus (HIV), viral hepatitis B and C, viral respiratory infections, vector-borne diseases such as malaria and leishmaniasis, food- and waterborne diseases including cholera, and an increased risk of antibiotic-resistant strains. In general, traditional epidemiological surveillance systems are not well-adapted to detecting emerging risks posed by climate change in the field of public health and climate refugees. Therefore, additional efforts will be needed to improve risk identification and assessment as it pertains to the different levels of climate management, be it environmental changes, population health impact, or health system status. Physicians should be trained to use efficient diagnostic and therapeutic management methods for these pathologies whose low frequency in our nonendemic regions make these pathologies exotic. 

The conditions in which refugees and migrants travel can acutely exacerbate or cause a life-threatening deterioration of their health. Elderly people and children are particularly vulnerable. Complications can result from physical injuries, loss of access to medication or devices, loss of prescriptions, lack of access to health care services leading to prolongation of disruption of treatment, and degradation of living conditions (loss of shelter, shortages of water and regular food supplies, and lack of income add to physical and psychological strain). Additionally, interruption of care due to destruction of health infrastructure, disruption of medical supplies, and the absence of health care providers, including interruption of power supplies or safe water resources, especially for people with end-stage renal failure who require dialysis, must be anticipated. A fundamental principle to remember, protecting the health of migrants, especially the most vulnerable populations, must be a priority. 

### 5.3. Outreach to Assist Less Resourced Nations

Developing or resource-poor countries are the first victims of climate change, while their role in producing greenhouse gases is the weakest. A recent report [80] predicts that climate change will create economic winners and losers at both the individual and sectoral level, but developing countries will suffer disproportionately from rise in temperatures since they are situated in relatively hot climates. Within developing countries, the poor would likely be the most heavily affected by climate change. It is essential to support them in climate change prevention and response projects. The vulnerability of these countries is linked to a lack of financial resources, adequate and efficient technology, and effective institutions. More assistance for developing countries needing transfer of knowledge, technology and financial resources, and interventions promoting environmental/climate protection would help to promote adaption strategies among developing countries. 

## 6. Reducing the Carbon Footprint of Health Services and EDs in Particular 

Hospitals produce significant amounts of greenhouse gases. The health care sector was responsible for eight percent of total U.S. greenhouse gas emissions, as they are heavy energy consumers [81]. The U.S. Department of Health and Human Services (HHS) outlined a wide range of strategies in its Strategic Sustainability Performance Plan (SSPP) focused on improving the energy efficiency of HHS-owned facilities [82]: reducing the production of greenhouse gases by limiting energy consumption and reducing the consumption of products with a high carbon footprint; the construction of buildings respecting the standards of sustainability, promoting clean and renewable energy and optimizing water-use efficiency and management; and fleet management by promoting the use of alternative fuel, electric, and zero-emission vehicles. Sustainability is also promoted by introducing new policies to achieve reduction in fuel use by decreasing the number of total vehicles in the motor pool, properly distributing newly acquired alternative fuel vehicles and encouraging ridesharing for employees who utilize fleet resources, sustainable acquisition models, pollution prevention and waste reduction, performance contracting, electronic stewardship and data centers, and to promote climate change resilience by incorporating climate adaptation and resilience solutions into the design of facilities through master planning. 

## 7. Conclusions

Climate change leads to an increased frequency of violent phenomena and natural disasters. This will result in the repeated management of crises with serious deaths and injuries. This will also lead to the emergence of epidemics and an increase in the flow of patients requiring emergency services. Moreover, climate change will be responsible for a change in the epidemiology of pathologies usually encountered in emergency health settings. It will also be responsible for the emergence of new diseases and the re-emergence of extinct diseases. Emergency departments should anticipate the increase in consultations because of climate changes and acute variations in temperature. Educational institutions and health and human service organizations must commit to increasing access to disaster simulation-based education and exercises, in an effort to enhance disaster preparedness of emergency caregivers.

## References

[1] Solomon S., Qin D., Manning M., Chen Z., Marquis M., Averyt M., Miller H.L. (2016). Climate Change 2007: The Physical Science Basis. Contribution of Working Group I to the Fourth Assessment Report of the Intergovernmental Panel on Climate Change.

[2] Cook J., Oreskes N., Doran P.T., Anderegg W.R.L., Verheggen B., Maibach E.W., Carlton J.S., Lewandowsky S., Skuce A.G., Green S.A. (2012). Consensus on consensus: A synthesis of consensus estimates on human-caused global warming. Environ. Res. Lett..

[3] Woodwarda A., Porterb J.R. (2016). Food, hunger, health, and climate change. Lancet.

[4] Wuebbles D.J., Easterling D.R., Hayhoe K., Knutson T., Kopp R.E., Kossin J.P., Kunkel K.E., LeGrande A.N., Mears C., Sweet W.V., Wuebbles D.J., Fahey D.W., Hibbard K.A., Dokken D.J., Stewart B., Maycock T.K. (2017). Our globally changing climate. Climate Science Special Report: Fourth National Climate Assessment.

[5] Casalino E., Choquet C., Wargon M., Curac S., Duchateau F.X., Revue E., Hellman R. (2017). Changement climatique: Proposition d’une cartographie des risques pour la santé et la médecine d’urgence en France. Ann. Fr. Med. Urgence.

[6] Wenju C., Simon B., Matthieu L., van Rensh P., Collins M., Vecchi G., Timmermann A., Santoso A., McPhaden M.J., Wu L. (2014). Increasing frequency of extreme El Niño events due to greenhouse warming. Nat. Clim. Chang..

[7] Ebi K., Exuzides K., Lau E., Kelsh M., Barnston A. (2004). Weather changes associated with hospitalizations for cardiovascular diseases and stroke in California, 1983–1998. Int. J. Biometeorol..

[8] Costello A., Abbas M., Allen A., Ball S., Bell S., Bellamy R., Friel S., Groce N., Johnson A., Kett M. (2009). Managing the health effects of climate change: Lancet and University College London Institute for Global Health Commission. Lancet.

[9] International Organization for Migration (2016). Migration and Climate Change. https://www.iom.int/migration-and-climate-change.

[10] Springmann M., Mason-D’Croz D., Robinson S., Garnett T., Godfray H.C., Gollin D., Rayner M., Ballon P., Scarborough P. (2016). Global and regional health effects of future food production under climate change: A modelling study. Lancet.

[11] Farajzadeh M., Darand M. (2009). Analyzing the influence of air temperature on cardiovascular, respiratory and stroke mortality in Tehran, Iran. J. Environ. Health Sci. Eng..

[12] Schwartz J., Samet J.M., Patz J.A. (2004). Hospital admissions for heart disease. Epidemiology.

[13] The Eurowinter Group (1997). Cold exposure and winter mortality from ischaemic heart disease, cerebrovascular disease, respiratory disease, and all causes in warm and cold regions of Europe. Lancet.

[14] Greenberg J.H., Bromberg J., Reed C.M., Gustafson T.L., Beauchamp R.A. (1983). The epidemiology of heat-related deaths, Texas–1950, 1970–1979, and 1980. Am. J. Public Health.

[15] Basu R., Samet J.M. (2002). Relation between elevated ambient temperature and mortality: A review of the epidemiologic evidence. Epidemiol. Rev..

[16] Giang P.N., Dung Do V., Bao Giang K., Van Vinh H., Rocklöv J. (2014). The effect of temperature on cardiovascular disease hospital admissions among elderly people in Thai Nguyen Province, Vietnam. Glob. Health Action.

[17] Zhang Y., Wang S.G., Ma Y.X., Shang K.Z., Cheng Y.F., Li X., Ning G.C., Zhao W.J., Li N.R. (2015). Association between ambient air pollution and hospital emergency admissions for respiratory and cardiovascular diseases in Beijing: A time series study. Biomed. Environ. Sci..

[18] D’Amato G., Holgate S.T., Pawankar R., Ledford D.K., Cecchi L., Al-Ahmad M., Al-Enezi F., Al-Muhsen S., Ansotegui I., Baena-Cagnani C.E. (2015). Meteorological conditions, climate change, new emerging factors, and asthma and related allergic disorders. A statement of the World Allergy Organization. World Allergy Organ. J..

[19] Pirozzi C.S., Jones B.E., VanDerslice J.A., Zhang Y., Paine R., Dean N.C. (2018). Short-Term Air Pollution and Incident Pneumonia: A Case-Crossover Study. Ann. Am. Thorac. Soc..

[20] Glaser J., Lemery J., Rajagopalan B., Diaz H.F., García-Trabanino R., Taduri G., Madero M., Amarasinghe M., Abraham G., Anutrakulchai S. (2016). Climate Change and the Emergent Epidemic of CKD from Heat Stress in Rural Communities: The Case for Heat Stress Nephropathy. Clin. J. Am. Soc. Nephrol..

[21] McMichael A.J., Wilkinson P., Kovats R.S., Pattenden S., Hajat S., Armstrong B., Vajanapoom N., Niciu E.M., Mahomed H., Kingkeow C. (2008). International study of temperature, heat and urban mortality: The ‘ISOTHURM’ project. Int. J. Epidemiol..

[22] Barnett A.G., Dobson A.J., McElduff P., Salomaa V., Kuulasmaa K., Sans S., WHO MONICA Project (2005). Cold periods and coronary events: An analysis of populations worldwide. J. Epidemiol. Commun. Health.

[23] Anderson B.G., Bell M.L. (2009). Weather-related mortality: How heat, cold, and heat waves affect mortality in the United States. Epidemiology.

[24] Goldberg M.S., Gasparrini A., Armstrong B., Valois M.F. (2011). The short-term influence of temperature on daily mortality in the temperate climate of Montreal, Canada. Environ. Res..

[25] Mastrangelo G., Fedeli U., Visentin C., Milan G., Fadda E., Spolaore P. (2007). Pattern and determinants of hospitalization during heat waves: An ecologic study. BMC Public Health.

[26] Belmin J., Auffray J.-C., Berbezier C., Boirin P., Mercier S., de Reviers B., Golmard J.L. (2007). Level of dependency: A simple marker associated with mortality during the 2003 heatwave among French dependent elderly people living in the community or in institutions. Age Ageing.

[27] Huang W., Kan H., Kovats S. (2010). The impact of the 2003 heat wave on mortality in Shanghai, China. Sci. Total Environ..

[28] Goggins W.B., Chan E.Y., Yang C., Chong M. (2013). Associations between mortality and meteorological and pollutant variables during the cool season in two Asian cities with sub-tropical climates: Hong Kong and Taipei. Environ. Health..

[29] Wang X.Y., Barnett A.G., Yu W., FitzGerald G., Tippett V., Aitken P., Neville G., McRae D., Verrall K., Tong S. (2012). The impact of heatwaves on mortality and emergency hospital admissions from non-external causes in Brisbane, Australia. Occup. Environ. Med..

[30] Turner L.R., Connell D., Tong S. (2010). Exposure to hot and cold temperatures and ambulance attendances in Brisbane, Australia: A time-series study. BMJ Open.

[31] Bai L., Ding G., Gu S., Bi P., Su B., Qin D., Xu G., Liu Q. (2014). The effects of summer temperature and heat waves on heat-related illness in a coastal city of China, 2011–2013. Environ. Res..

[32] European Commission (2016). Climate Change. Flooding in Europe: Health Risks. http://ec.europa.eu/health/climate_change/extreme_weather/flooding/index_en.htm.

[33] World Health Organization (2016). Climate Change and Health. http://www.who.int/mediacentre/factsheets/fs266/en/.

[34] LaKind J.S., Overpeck J., Breysse P.N., Backer L., Richardson S.D., Sobus J., Sapkota A., Upperman C.R., Jiang C., Beard C.B. (2016). Exposure science in an age of rapidly changing climate: Challenges and opportunities. J. Expo. Sci. Environ. Epidemiol..

[35] Medlock J.M., Leach S.A. (2015). Effect of climate change on vector-borne disease risk in the UK. Lancet Infect. Dis..

[36] Pijnacker R., Goris M.G., Te Wierik M.J., Broens E.M., van der Giessen J.W., de Rosa M., Wagenaar J.A., Hartskeerl R.A., Notermans D.W., Maassen K. (2016). Marked increase in leptospirosis infections in humans and dogs in The Netherlands, 2014. Euro Surveill..

[37] Hansen A., Cameron S., Liu Q., Sun Y., Weinstein P., Williams C., Han G.S., Bi P. (2015). Transmission of haemorrhagic fever with renal syndrome in China and the role of climate factors: A review. Int. J. Infect. Dis..

[38] Campbell-Lendruma D., Corvalána C., Neiraa M. (2007). Global climate change: Implications for international public health policy. Bull. World Health Organ..

[39] Piperaki E.T., Daikos G.L. (2016). Malaria in Europe: Emerging threat or minor nuisance?. Clin. Microbiol. Infect..

[40] Poulakou G., Bassetti M., Timsit J.F. (2016). Critically ill migrants with infection: Diagnostic considerations for intensive care physicians in Europe. Intens. Care Med..

[41] European Academies Science Advisory Council (2007). Impact of Migration on Infectious Diseases in Europe. http://www.easac.eu/fileadmin/PDF_s/reports_statements/Migration.pdf.

[42] Gordon C.A., McManus D.P., Jones M.K., Gray D.J., Gobert G.N. (2016). The increase of exotic zoonotic helminth infections: The impact of urbanization, climate change and globalization. Adv. Parasitol..

[43] Mellor J.E., Levy K., Zimmerman J., Elliott M., Bartram J., Carlton E., Clasen T., Dillingham R., Eisenberg J., Guerrant R. (2016). Planning for climate change: The need for mechanistic systems-based approaches to study climate change impacts on diarrheal diseases. Sci. Total Environ..

[44] Carlton E.J., Woster A.P., DeWitt P., Goldstein R.S., Levy K. (2016). A systematic review and meta-analysis of ambient temperature and diarrhoeal diseases. Int. J. Epidemiol..

[45] Na W., Lee K.E., Myung H.N., Jo S.N., Janq J.Y. (2016). Incidences of Waterborne and Foodborne Diseases after Meteorologic Disasters in South Korea. Ann. Glob. Health.

[46] Bezirtzoglou C., Dekas K., Charvalos E. (2011). Climate changes, environment and infection: Facts, scenarios and growing awareness from the public health community within Europe. Anaerobe.

[47] Young I., Smith B.A., Fazil A. (2015). A systematic review and meta-analysis of the effects of extreme weather events and other weather-related variables on Cryptosporidium and Giardia in fresh surface waters. J. Water Health.

[48] Crowley R.A. (2016). Climate change and health: A position paper of the American College of Physicians. Ann. Intern. Med..

[49] Fountoulakis K.N., Chatzikosta I., Pastiadis K., Zanis P., Kawohl W., Kerkhof A.J., Navickas A., Höschl C., Lecic-Tosevski D., Sorel E. (2016). Relationship of suicide rates with climate and economic variables in Europe during 2000–2012. Ann. Gen. Psychiatry.

[50] Fountoulakis K.N., Savopoulos C., Zannis P., Apostolopoulou M., Fountoukidis I., Kakaletsis N., Kanellos I., Dimellis D., Hyphantis T., Tsikerdekis A. (2016). Climate change but not unemployment explains the changing suicidality in Thessaloniki Greece (2000–2012). J. Affect. Disord..

[51] Oudin A., Åström D.O., Asplund P., Steingrimsson S., Szabo Z., Carlsen H.K. (2018). The association between daily concentrations of air pollution and visits to a psychiatric emergency unit: A case-crossover study. Environ. Health..

[52] Rataj E., Kunzweiler K., Garthus-Niegel S. (2016). Extreme weather events in developing countries and related injuries and mental health disorders—A systematic review. BMC Public Health.

[53] IDMC (Internal Displacement Monitoring Center) (2017). Global Disaster Displacement Risk. A Baseline for Future Work. http://www.internal-displacement.org/assets/publications/2017/201710-IDMC-Global-disaster-displacement-risk.pdf.

[54] Pachauri R.K., Meyer L.A., Intergovernmental Panel on Climate Change (IPCC) (2014). Climate Change 2014: Synthesis Report.

[55] Luber G., Prudent N. (2009). Climate change and human health. Trans. Am. Clin. Climatol. Assoc..

[56] CDC (Centers for Disease Control and Prevention) (2016). Climate Effects on Health. http://www.cdc.gov/climateandhealth/effects/.

[57] D’Amato G., Pawankar R., Vitale C., Lanza M., Molino A., Stanziola A., Sanduzzi A., Vatrella A., D’Amato M. (2016). Climate change and air pollution: Effects on respiratory allergy. Allergy Asthma Immunol. Res..

[58] Sherbakov T., Malig B., Guirguis K., Gershunov A., Basu R. (2018). Ambient temperature and added heat wave effects on hospitalizations in California from 1999 to 2009. Environ. Res..

[59] Banholzer S., Kossin J., Donner S., Zommers Z., Singh A. (2014). The Impact of Climate Change on Natural Disasters. Reducing Disaster: Early Warning Systems for Climate Change.

[60] Intergovernmental Panel on Climate Change. https://www.ipcc.ch/news_and_events/outreach.shtml.

[61] (2014). U.S. Department of Health and Human Services (HHS) Climate Adaptation Plan. https://www.hhs.gov/sites/default/files/about/sustainability/2014-climate-change.pdf.

[62] World Health Organization (2018). Climate Change and Health. http://www.who.int/news-room/fact-sheets/detail/climate-change-and-health.

[63] Ren J., Li B., Yu D., Liu J., Ma Z. (2016). Approaches to prevent the patients with chronic airway diseases from exacerbation in the haze weather. J. Thorac. Dis..

[64] (2017). World Association for Disaster and Emergency Medicine. WADEM Climate Change Position Statement. Prehosp. Disaster Med..

[65] Tseng C.H., Lu L.C., Lan S.H., Hsieh Y.P., Lan S.J. (2017). Relationship between emergency care utilization, ambient temperature, and the pollution standard index in Taiwan. Int. J. Environ. Health Res..

[66] World Health Organization (2016). 2015 Report: WHO’s Work in Emergency Risk and Crisis Management. http://apps.who.int/iris/bitstream/handle/10665/251551/WHO-WHE-ERM-EXT-2016.4-eng.pdf?sequence=1.

[67] Ahalt V., Argon N.T., Ziya S., Strickler J., Mehrotra A. (2018). Comparison of emergency department crowding scores: A discrete-event simulation approach. Health Care Manag. Sci..

[68] Chiu I.M., Lin Y.R., Syue Y.J., Kung C.T., Wu K.H., Li C.J. (2018). The influence of crowding on clinical practice in the emergency department. Am. J. Emerg. Med..

[69] Kavanagh K., Shields D., Staunton P. (2017). 40 ED crowding: The acceptability of dysfunction. Emerg. Med. J..

[70] Chang A.M., Cohen D.J., Lin A., Augustine J., Handel D.A., Howell E., Kim H., Pines J.M., Schuur J.D., McConnell K.J. (2018). Hospital Strategies for Reducing Emergency Department Crowding: A Mixed-Methods Study. Ann. Emerg. Med..

[71] Pan C.L., Chiu C.W., Wen J.C. (2014). Adaptation and promotion of emergency medical service transportation for climate change. Medicine (Baltimore).

[72] Van Minh H., Tuan Anh T., Rocklöv J., Bao Giang K., Trang le Q., Sahlen K.G., Nilsson M., Weinehall L. (2014). Primary healthcare system capacities for responding to storm and flood-related health problems: A case study from a rural district in central Vietnam. Glob. Health Action.

[73] Williams H., Downes E. (2017). Development of a Course on Complex Humanitarian Emergencies: Preparation for the Impact of Climate Change. J. Nurs. Scholarsh..

[74] Labrague L.J., Hammad K., Gloe D.S., McEnroe-Petitte D.M., Fronda D.C., Obeidat A.A., Leocadio M.C., Cayaban A.R., Mirafuentes E.C. (2018). Disaster preparedness among nurses: A systematic review of literature. Int. Nurs. Rev..

[75] VanDevanter N., Raveis V.H., Kovner C.T., McCollum M., Keller R. (2017). Challenges and Resources for Nurses Participating in a Hurricane Sandy Hospital Evacuation. J. Nurs. Scholarsh..

[76] Park H.Y., Kim J.S. (2017). Factors influencing disaster nursing core competencies of emergency nurses. Appl. Nurs. Res..

[77] Lam R.P.K., Balsari S., Hung K.K.C., Hsiao K.H., Leung L.P., Leaning J. (2017). How Do Doctors and Nurses in Emergency Departments in Hong Kong View Their Disaster Preparedness? A Cross-Sectional Territory-Wide Online Survey. Disaster Med. Public Health Prep..

[78] Veenema T.G., Lavin R.P., Griffin A., Gable A.R., Couig M.P., Dobalian A.C. (2017). All to Action: The Case for Advancing Disaster Nursing Education in the United States. J. Nurs. Scholarsh..

[79] The United Nation Refugee Agency (2018). Climate Change and Disasters. http://www.unhcr.org/climate-change-and-disasters.html.

[80] United Nations Climate change (2017). Developing Countries Need Urgent Support to Adapt to Climate Change. https://unfccc.int/news/developing-countries-need-urgent-support-to-adapt-to-climate-change.

[81] Climate for Health (2016). How Hospitals Can Reduce Their Carbon Footprints. http://climateforhealth.org/blog/how-hospitals-can-reduce-their-carbon-footprints.

[82] U.S. Department of Health and Human Services (HHS) (2017). U.S. Department of Health and Human Services Strategic Sustainability Performance Plan July 2017. https://www.hhs.gov/sites/default/files/2017-HHS-Strategic-Sustainability-Performance-Plan_0.pdf.

